# Five-aza-2′-deoxycytidine-induced hypomethylation of *cholesterol 25-hydroxylase* gene is responsible for cell death of myelodysplasia/leukemia cells

**DOI:** 10.1038/srep16709

**Published:** 2015-11-18

**Authors:** Takayuki Tsujioka, Akira Yokoi, Yoshitaro Itano, Kentaro Takahashi, Mamoru Ouchida, Shuichiro Okamoto, Toshinori Kondo, Shin-ichiro Suemori, Yumi Tohyama, Kaoru Tohyama

**Affiliations:** 1 Department of Laboratory Medicine, Kawasaki Medical School, Okayama 701-0192, Japan; 2 Eisai Co., Ltd., Tsukuba, Ibaraki, 300-2635, Japan; 3 Department of Anesthesiology, Kawasaki Medical School, Okayama 701-0192, Japan; 4 Department of Molecular Genetics, Okayama University Graduate School of Medicine, Dentistry and Pharmaceutical Sciences, Okayama 700-8558, Japan; 5 Division of Hematology, Department of Internal Medicine, Kawasaki Medical School, Okayama 701-0192, Japan; 6 Division of Biochemistry, Faculty of Pharmaceutical Sciences, Himeji Dokkyo University, Hyogo 670-8524, Japan

## Abstract

DNA methyltransferase inhibitors (DNMT inhibitors) are administered for high-risk MDS, but their action mechanisms are not fully understood. Hence, we performed a genome-wide DNA methylation assay and focused on *cholesterol 25-hydroxylase* (*CH25H*) among the genes whose expression was up-regulated and whose promoter region was hypomethylated after decitabine (DAC) treatment *in vitro*. CH25H catalyzes hydroxylation of cholesterol and produces 25-hydroxycholesterol (25-OHC). Although *CH25H* mRNA expression level was originally low in MDS/leukemia cell lines, exposure to DNMT inhibitors enhanced *CH25H* mRNA expression. The promoter region of *CH25H* was originally hypermethylated in HL-60 and MDS-L cells, but DAC treatment induced their hypomethylation together with increased *CH25H* mRNA expression, activation of *CH25H*-oxysterol pathway, 25-OHC production and apoptotic cell death. We further confirmed that normal CD34-positive cells revealed hypomethylated status of the promoter region of *CH25H* gene. *CH25H*-knockdown by transfection of shRNA lentiviral vector into the cell lines partially protected the cells from DAC-induced cell death. Exogenous addition of 25-OHC suppressed leukemic cell growth. The present study raises a possibility that DNMT inhibitors activate *CH25H*-oxysterol pathway by their hypomethylating mechanism and induce leukemic cell death. Further investigations of the promoter analysis of *CH25H* gene and therapeutic effects of DNMT inhibitors on MDS/leukemia will be warranted.

The myelodysplastic syndromes (MDS) are a group of acquired hematopoietic disorders characterized by cytopenia(s) and dysplasia as a result of clonal growth of pathological stem cells showing ineffective hematopoiesis, and by the increased risk of progression to acute myeloid leukemia (AML)^1^,^2^. Hematopoietic stem cell transplantation (HSCT) is the only potentially curative treatment, but HSCT is restricted to a small population of MDS patients owing to the factors such as advanced ages, concomitant comorbidities and donor availability^3^. DNA methyltransferase(DNMT) inhibitors, 5-azacytidine (azacitidine; AZA) and 5-aza-2′-deoxycytidine (decitabine; DAC) have recently been used as chemotherapeutic agents for MDS patients who are not eligible for HSCT^4^,^5^,^6^,^7^,^8^,^9^. Notably, the treatment with AZA significantly increased overall survival in patients with high-risk MDS as compared with conventional treatments (clinical trial: AZA-001)^10^. However, the mechanism of action of DNMT inhibitors has not been clearly defined^11^,^12^,^13^,^14^,^15^,^16^.

We previously investigated the effects of DNMT inhibitors on the MDS cell lines established in our laboratory, and demonstrated that DAC-induced cell death was preceded by a DNA damage response via a p53-independent pathway^17^. Furthermore, we researched some genes involved in the mechanism of action of DAC by a gene expression profiling.

In this study, we performed a genome-wide DNA methylation assay and consequently focused on *cholesterol 25-hydroxylase* (*CH25H*) among the genes whose expression was up-regulated and whose promoter region was hypomethylated after DAC treatment *in vitro*. CH25H is an important enzyme which catalyzes the hydroxylation of cholesterol and produces 25-hydroxycholesterol(25-OHC)^18^. Twenty-five-OHC, one of the endogenous oxysterols, has been reported to inhibit cell growth in various tumor cells^19^,^20^,^21^,^22^. We confirmed that the promoter region of *CH25H* in the two cell lines was originally hypermethylated and DAC treatment induced their hypomethylation which was associated with increased *CH25H* mRNA expression, activation of *CH25H*-oxysterol pathway, 25-OHC production and apoptotic cell death. We further confirmed that normal CD34-positive cells present hypomethylated status of the promoter region of *CH25H.* Therefore, we propose a hypothesis that *CH25H* is one of the candidate genes whose methylation status is related to myeloid neoplasms and one of the target genes of DNMT inhibitors.

## Materials and Methods

### Reagents

Five-aza-2′-deoxycytidine (decitabine; DAC, Sigma-Aldrich Co, St. Louis, MO, USA), 5-azacytidine (azacitidine; AZA, Wako Pure Chemical Industries, Ltd, Osaka, Japan) and cytosine arabinoside (cytarabine; ara-C, Nippon Shinyaku Co., Ltd, Kyoto, Japan) were dissolved in distilled water and stored at −20 °C. For the *in vitro* studies, we used each agent at the concentrations of 1 to 10^4^  nM. They were added to the cultured cells daily without changing the culture medium.

Twenty-five-hydroxycholesterol (cholest-5-ene-3β,25-diol; 25-OHC), and a CH25H enzyme inhibitor, desmosterol (5,24-cholestadien-3β-ol)^19^ were purchased from Sigma Aldrich. Co (St. Louis, MO, USA). Twenty-four-hydroxycholesterol (cholest-5-ene-3ß,24(S)-diol; 24-OHC) and 27-hydroxycholesterol (cholest-5-ene-3β,27-diol; 27-OHC) were purchased from Avanti Polar Lipids, Inc. (Alabaster, AL, USA). They were dissolved in ethanol and stored at −20 °C.

### Cell lines and culture

A myelodysplastic cell line, MDS92 was established from the bone marrow of a patient with MDS^23^. This cell line proliferated in the presence of interleukin-3 (IL-3) or granulocyte-macrophage colony-stimulating factor (GM-CSF) with a tendency to mature gradually^24^,^25^.

MDS-L and MDS92T cell lines were established independently each other from the long-term culture of parental MDS92. MDS-L cells showed blastic morphology and were positive for CD34, c-Kit, HLA-DR, CD13 and CD33^26^. MDS92T cell line consisted of immature myeloid cells with indented nucleus and was negative for CD34 exclusively.

MDS92, MDS-L and MDS92T cells were maintained in RPMI1640 medium supplemented with 10% fetal bovine serum, 50 μM 2-mercaptoethanol, 2.0 mM L-glutamine and 100 U/ml IL-3. A human myeloid leukemia cell line, HL-60, a blastic cell line from chronic myelogenous leukemia, K562 and a diffuse histiocytic lymphoma cell line, U937 were also used.

### Primary cells

Five human normal bone marrow CD34-positive progenitor cells were purchased from LONZA Group Ltd, Basel, Switzerland and cultured with the serum-free medium with recombinant cytokines for myelopoiesis of hematopoietic progenitor cells, STEM ALPHA, AG (FUNAKOSHI, Tokyo, Japan). Pathological bone marrow samples were obtained from untreated patients with MDS or AML at the Department of Hematology, Kawasaki Medical School Hospital after obtaining informed consent from each patient. We performed all experiments in accordance with the Declaration of Helsinki and approved guidelines. The usage of patient samples was approved by the Ethical Committee of Kawasaki Medical School. The mononuclear cell fraction was isolated from the bone marrow samples by Ficoll-Hypaque density centrifugation as manufacturer’s protocols and CD34-positive fraction was purified by the magnetic beads method with anti-CD34 monoclonal antibody. The purity of this fraction was more than 95%.

### Cell growth assay and MTT assay

Cell growth was assessed by counting the number of living cells after trypan blue staining. The morphological assessment was performed with May-Gruenwald Giemsa-stained cytospin slides. Cell suspensions were plated into 96-well plates in the presence of the drug or solvent alone, incubated as above at 37 °C for 3–7days, and analyzed by the 3-(4,5-dimethythiazol-2-yl)-2,5-diphenyl tetrazolium bromide (MTT) assay as previously described^17^.

### Apoptosis assay

Apoptosis was examined using Annexin V Apoptosis Detection Kit (BD Pharmingen, San Diego, CA, USA), and all samples were analyzed with FACS Calibur flow cytometer and CellQuest software (Becton Dickinson, Franklin Lakes, NJ, USA)^27^,^28^.

### Genome-wide methylation sequencing analysis (MethylC-seq analysis)

The detailed information of this analysis is described in Supplementary Materials and methods (Ref. 29-32, 30, 31, 32).

### Quantitative real-time reverse transcription PCR

Quantitative real-time reverse transcription PCR (q-PCR) was performed with Applied Biosystems StepOne Plus^TM^ Real time PCR system (Life Technologies Japan Ltd, Tokyo). Total cellular RNA (3–5 μg) was used to synthesize cDNA by using Ready-To-Go T primed First-Strand Kit (GE Healthcare UK Ltd, Amersham Place, Little Chalfont, Buckinghamshire HP7 9NA, UK) in a final volume of 33 μl. Five μl of 1:10 diluted cDNA reactions was used as input for each of the real-time quantitative PCR reactions by using SYBR Premix Ex Taq^TM^ (Takara Bio Inc, Shiga, Japan). Initial denaturation at 95 °C for 20 s was followed by 40 cycles of a denaturation step at 95 °C for 3 s and an annealing and extension step at 60 °C for 30 s. Primers were designed by using Primer 3 v 4.0.0 software (Initial development of Primer3 was funded by Howard Hughes Medical Institute and by the National Institutes of Health, National Human Genome Research Institute). Since human *CH25H* gene spans one exon, we used total RNA treated with DNase I (Qiagen, Tokyo, Japan) so that the experimental results are not compromised through genomic DNA contamination^33^.

*CH25H* (NM_003956) forward primer was 5′-TGGCAACGCAGTATATGAGC-3′ and its reverse primer was 5′-AGGGAAGTTGTAGCCGGAGT-3′. Human housekeeping gene *RPL27* (NM_000988) was used as endogenous control^30^. Primers for *RPL27* were as follows: forward: 5′-CTCTGGTGGCTGGAATTGAC-3′ and reverse: 5′-AAACCGCAGTTTCTGGAAGA-3′.

The copy ratio of *CH25H* to *RPL27* was calculated and indicated as a quantification of *CH25H* expression in several experiments.

### Gene expression profiling and the gene set enrichment analysis (GSEA)

The detailed information of these experiments is described in Supplementary Materials and methods (Ref.^35-36^, ^36^).

### Methylation analysis of the promoter region with bisulfite sequencing

Genomic DNA was extracted using SepaGene DNA extraction Kit (Sanko Junyaku, Tokyo, Japan) and chemically modified with sodium bisulfite using MethylEasy^TM^ Xceed Rapid DNA Bisulfite Modification Kit (Human Genetic Signatures Pty Ltd, North Ryde, Australia), which converts all unmethylated cytosines to uracils, whereas methylated cytosines remain unchanged. Analysis of the obtained sequences confirmed a complete bisulfite reaction in all samples^33^. Then, the 1700 bp upstream sequence of the transcription initiation site which might include the promoter region was divided into six regions and they were defined as F1 to F6, respectively.

Bisulfite-treated DNA was amplified in PCR with *cholesterol 25-hydroxylase* (*CH25H*)-specific primers F1-6. Primers for each region of *CH25H* and the PCR product sizes are shown in supplementary Table S1.

PCR was performed in 50 μl of reaction mixture with 0.4 μM of each primer, 5 ng of genomic DNA, 1 × EpiTaqPCR buffer (Mg2 + free), 0.3 mM of each deoxynucleotide triphosphate, 2.5 mM MgCl_2_ and 1.25 U of EpiTaq HS (Takara, Tokyo, Japan). Initial denaturation at 98 °C for 3 min was followed by 40 cycles of a denaturation step at 98 °C for 10 s, an annealing step at 55 °C for 30 s, an extension step at 72 °C for 1 min, and a final extension step of 72 °C for 7 min was added. The products were separated by electrophoresis on 1.5% agarose gel. PCR products were gel-purified using Wizard^R^SV Gel and PCR Clean-up System (Promega Co, Madison, USA) and ligated into the pCR^TM^II-TOPO^R^ vector (Invitrogen by Life Technologies, Tokyo, Japan) according to the manufacturer’s instructions. Independent plasmid clones were purified from several bacterial colonies. Five to ten clones per sample were sequenced using the Big Dye terminator ver 3.1 Cycle Sequencing Kit (Applied Biosystems) and ABI Prism 3130 Genetic Analyzer (Applied Biosystems, Tokyo, Japan).

### Plasmids and transfection

To inhibit *CH25H* gene expression, a vector for short-hairpin RNA (shRNA) against *CH25H* incorporated in pLKO.1-puro (Sigma-Aldrich, Mission shRNA code: TRCN115963) was transfected into HL-60 and MDS-L cells by ViraPower lentiviral Directional TOPO Expression kit (Invitrogen)^37^ and positive clones were selected with 2 μg/ml puromycin. Likewise, we introduced the control vector (Sigma-Aldrich, Mission TurboGFP^TM^ shRNA Control Vector) into HL-60 and MDS-L cells.

### Ultraperformance liquid chromatography coupled with Q-TOF mass spectrometry (UPLC/Q-TOF MS) and gas chromatography-mass spectrometry (GC-MS)

The detailed information of this analysis is described in Supplementary Materials and methods (Ref.^38-39^, ^39^).

### Statistical analyses

All results are shown as the mean values with ranges. Comparisons between the groups were done using the Dunnett’s and Scheffe tests. Differences were considered statistically significant if *p*-values were less than 0.05. These analyses were carried out using SPSS for Windows version 14.0.

## Results

### Decitabine (DAC) treatment leads to up-regulation of *cholesterol 25-hydroxylase (CH25H)* gene expression in MDS-L cell line

We previously explored the molecular pathways affected by DAC using the gene expression profiling of MDS-L cells treated with 4 nM DAC for 7 days^17^. Genes whose expression changed by more than 2-fold after DAC treatment were defined as regulated genes. 956 genes were up-regulated, and 461 genes were down-regulated in MDS-L cells treated with DAC (4 nM) as compared with the control. In the present study, we further performed the genome-wide DNA methylation assay (MethylC-seq)^29^,^30^,^31^,^32^ and identified 13 genes whose mRNA expression was up-regulated more than 20-fold and whose CpG islands at the promotor region (around 1000 bp upstream sequence of the transcription initiation site) were hypomethylated more than 10% after DAC treatment as compared with the control (Table 1).

Next, we surveyed the genes involved in cell growth suppression, differentiation and apoptosis among these 13 genes and focused on *cholesterol 25-hydroxylase* (*CH25H*) *gene*. In DNA microarray analysis, the expression of *CH25H* in DAC-treated MDS-L cells increased more than 180-fold as compared with that of control cells, and the 1000 bp upstream sequence of the transcription initiation site in *CH25H* was dominantly demethylated 45% by DAC treatment (Table 1).

**Figure 1:**
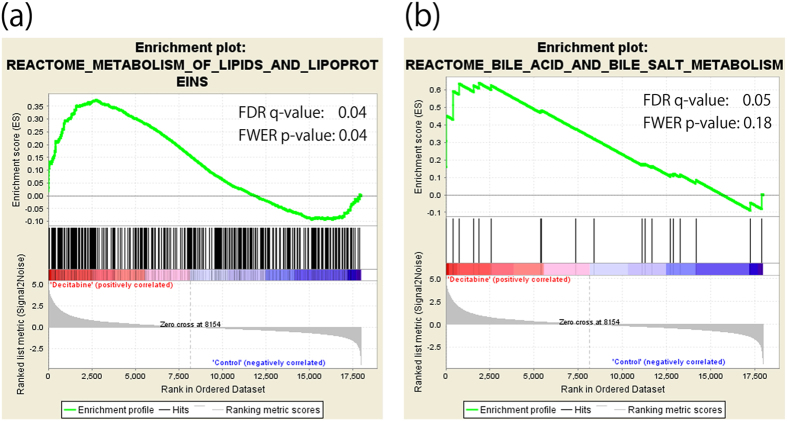
Activation of *CH25H*-related pathways in the gene set enrichment analysis (GSEA). DAC-treated or untreated MDS-L cells were harvested on day 7 in triplicate assays and the gene expression profiling data were used for GSEA by handling the GSEA software and the Molecular Signatures Database according to the references (35,36). Two gene sets strongly up-regulated by DAC treatment are presented: (**a**) metabolism of lipids and lipoproteins, and (**b**) bile acid and bile salt metabolism. Both gene sets contain *CH25H* as the most activated gene by DAC treatment (the heat map data are shown in Supplementary Figure S1). FDR q-value: false discovery rate; FWER p-value: family-wise error rate. The accumulation of the gene set is considered as statistically significant when both values are less than 0.25.

We further applied the data of DNA microarray analysis to the gene set enrichment analysis (GSEA) and found that two gene sets were strongly up-regulated by DAC treatment: ‘metabolism of lipids and lipoproteins’ and ‘bile acid and bile salt metabolism’. Both gene sets contained *CH25H* as the most activated gene by DAC treatment and its downstream genes, *CYP7B1* and *HSD3B7* (Fig. 1 and Supplementary Figure S1). CH25H catalyzes the hydroxylation of cholesterol and alters it to 25-hydroxycholeterol (25-OHC). Twenty-five-OHC is one of the natural oxysterols and is reported to inhibit the growth of several tumor cells^19^,^20^,^21^,^22^. The present data indicated that *CH25H*-oxysterol pathway is activated by DAC treatment in MDS-L cells.

### The treatment with DNMT inhibitors increases the expression of *CH25H* gene in MDS/leukemia cell lines

**Figure 2:**
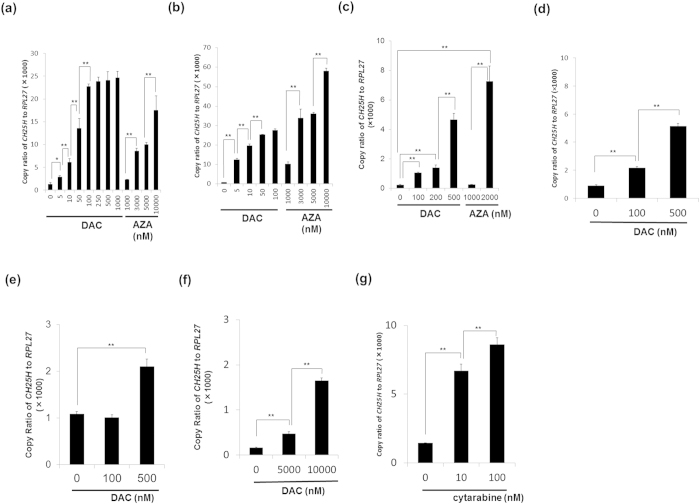
Induction of *CH25H* expression in MDS/leukemia cell lines treated with DNMT inhibitors. Cells were treated with DAC, AZA and cytarabine at various concentrations for 4 days or 7 days. Relative mRNA expression was analyzed by quantitative real-time PCR (described in Materials and Methods). Induction of *CH25H* mRNA expression in MDS-L treated with different concentrations of DAC or AZA for 4 days (**a**) and for 7 days (**b**), in HL-60 treated with different concentrations of DAC or AZA for 4 days (**c**), in MDS92T treated with different concentrations of DAC for 7 days (**d**) in U937 treated with different concentrations of DAC for 4 days (**e**), and in K562 treated with different concentrations of DAC for 4 days (**f,g**) Induction of *CH25H* mRNA expression in MDS-L treated with cytarabine for 4 days is shown. The data shown are the average ± SD of three independent experiments. *p < 0.05; **p < 0.01.

We examined *CH25H* mRNA expression level in five MDS/leukemia cell lines (HL-60, MDS-L, MDS92T, U937 and K562) by quantitative real-time PCR (q-PCR). The cells were treated with DAC or AZA at different concentrations as indicated for 4 or 7 days. Whereas *CH25H* mRNA expression level was originally low in all five cell lines, exposure of each cell line to DNMT inhibitors significantly increased the expression of *CH25H* (Fig. 2a–f).

*CH25H* mRNA expression in MDS-L cells treated with DAC for 4 or 7 days was increased in a dose-dependent and time-dependent manner (Fig. 2a,b). Likewise, the treatment with AZA for 4 or 7 days in MDS-L cells induced *CH25H* mRNA expression at high concentrations as compared with DAC (Fig. 2a,b). *CH25H* mRNA expression was also increased in HL-60 and the other cell lines treated with DAC or AZA (Fig. 2c–f). To confirm that up-regulation of *CH25H* mRNA in DAC-treated cell lines actually causes the production of 25-OHC, we tried to quantify 25-OHC in cell lysates or culture supernatants of DAC-treated cell lines by UPLC/Q-TOF MS and GC-MS. The analysis using the samples from less than several millions cells did not show any detectable amount of oxysterols, but we finally found a detectable amount of 25-OHC (46 ng/gram cell pellet) in a larger-scale sample of DAC-treated cells (10^9^ cells) in our assay system (detection sensitivity was 0.1 ng/g). In contrast, neither of the other oxysterols (24-OHC and 27-OHC) was detected in the same sample.

Next, we evaluated whether the increase of *CH25H* mRNA expression is specific to DNMT inhibitors or whether cytotoxic drugs such as cytarabine also induce it, and found that *CH25H* mRNA expression in MDS-L cells was increased after cytarabine treatment (Fig. 2g).

### The promoter region of *CH25H* is originally hypermethylated in both MDS-L and HL-60 cells

**Figure 3:**
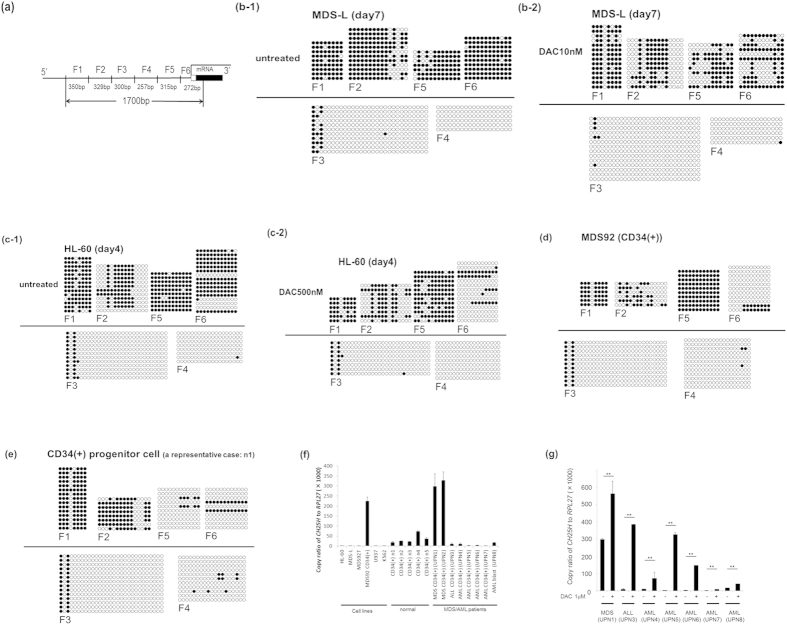
The methylation analysis in the promoter region of *CH25H* in the cell lines and normal and MDS/leukemia CD34-positive progenitor cells. (**a**) The schematic presentation of *CH25H* promoter sequence divided into 6 regions. Methylation status of independent plasmid clones derived from MDS-L (**b**), HL-60 (**c**) treated without or with DAC, CD34-positive cells of MDS92 (**d**) and a representative case (n1) of five normal CD34-positive progenitor cells (**e**). (● methylated cytosine, ○ unmethylated cytosine, △ not defined). (**f**) The expression of *CH25H* mRNA in normal CD34-positive progenitor cells (n = 5), six cell lines and CD34-positive or blastic cells from primary MDS/leukemia cells was compared. (**g**) Induction of *CH25H* mRNA expression is indicated in primary MDS/leukemia cells treated with 1 μM DAC for 4 days. In (**f,g**), The primary cases were categorized by FAB or WHO classification as follows: MDS (Refractory anemia: UPN1), MDS (Refractory cytopenia with multilineage dysplasia: UPN2), ALL(L2: UPN3), AML (M2: UPN4), AML (M7: UPN5), AML (M2: UPN6), AML (AML with myelodysplasia-related changes: UPN7), AML (M5b: UPN8). Purified CD34-positive cells in cases except UPN8 were used. Leukemia cells occupied more than 90% of bone marrow cells in case with UPN8. The data shown are the average ± SD of three independent experiments (*p < 0.05; **p < 0.01).

To further investigate the methylation status at the promoter region of *CH25H* in MDS-L and HL-60 cells, we performed the methylation analysis by bisulfite sequencing assay focusing on 1700 bp of the *CH25H* promoter region, by dividing the area into six regions, which spans from 1,647 bp-upstream of transcription initiation site to 32 codons of coding region in exon 1 (F1 to F6 regions in Fig. 3a and in Materials and methods). The DNA sequence in each region was amplified by PCR reaction using the primer sequences in the promoter region of *CH25H* (Supplementary Table S1). Each PCR product was ligated into the pCR^TM^II-TOPO^R^ vector and more than five clones per sample were sequenced. All primers do not contain any CpG sites. F6 region contains 32 codons of coding region in exon 1.

In untreated MDS-L and HL-60 cells, most CpG sites at F1, F2, F5 and F6 were methylated, whereas those of F3 and F4 remained unmethylated (Fig. 3b,c). In DAC-treated MDS-L and HL-60 cells, a considerable part of CpG sites at F2, F5 and F6 were demethylated (Fig. 3b,c). MDS92 cell line grows slowly with a tendency of maturation and appears to be less malignant than its descendant blastic subline, MDS-L and MDS92T.

Hence, we separated CD34-positive fraction from MDS92 for methylation analysis. In CD34-positive fraction of MDS92 cells, CpG sites at F5 of *CH25H* was hypermethylated, but not at F6 (Fig. 3d). *CH25H* mRNA expression in CD34-positive fraction of MDS92 cells was increased as compared with that of MDS-L and MDS92T (Fig. 3f). We further evaluated the methylation status of *CH25H* in CD34-positive progenitor cells derived from bone marrow of five healthy donors as a control. Strikingly, CpG sites at F5 and F6 in normal CD34-positive cells were hardly methylated (Fig. 3e). The mRNA expression in normal CD34-positive progenitor cells was generally increased as compared with the vigorous cell lines MDS-L, MDS92T, HL-60, U937 and K562 (p < 0.05) (Fig. 3f).

Next, we investigated the methylation status of *CH25H* F6 region in bone marrow CD34-positive cells derived from 3 MDS patients (UPN1, 2, 9) and one AML patient (UPN10), and found one case (UPN9) whose *CH25H* F6 region was hypermethylated out of 4 patients studied (Supplementary Figure S2). We could examine the expression of *CH25H* in two out of the above 4 cases and found that the expression of *CH25H* was increased in these cases (low-risk MDS: UPN1 and UPN2) (Fig. 3f). As both UPN1 and UPN2 revealed hypomethylation of *CH25H* F6 region (Supplementary Figure S2), it was consistent with increased *CH25H* mRNA expression. Regarding primary MDS/leukemia cells, CD34-positive or blastic cells indicated various levels of *CH25H* mRNA expression including two above described MDS cases showing marked *CH25H* expression (Fig. 3f). DAC treatment led to enhanced *CH25H* mRNA expression in most of primary leukemia cells studied (Fig. 3g).

### *CH25H*-knockdown by transfection of shRNA lentiviral vector in HL-60 and MDS-L cells partially protects the cells from DAC-induced cell death

**Figure 4:**
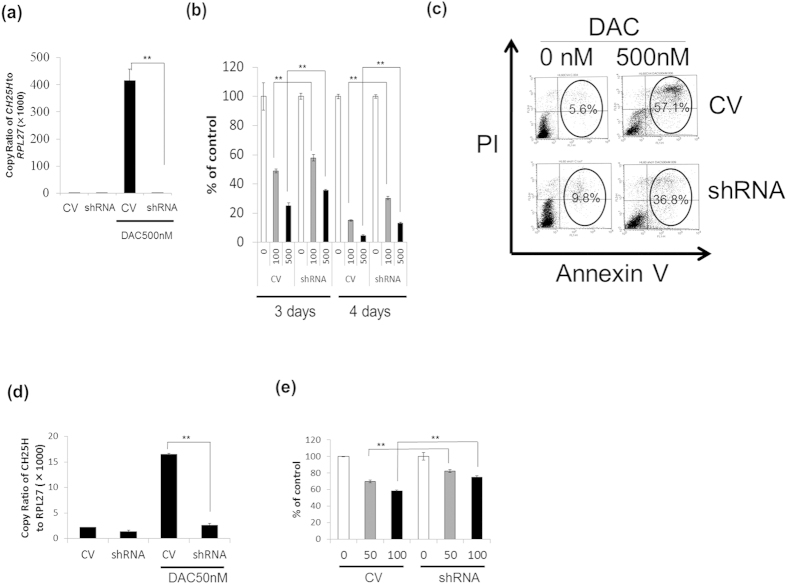
The role of *CH25H* in DAC-induced cell death. To investigate the role of *CH25H* in detail, we established *CH25H*-knockdown HL-60 cell clones (HL-60-*CH25H*shRNA) and MDS-L cell clones (MDS-L-*CH25H*shRNA) by transfecting shRNA-*CH25H* into each cell line using a lentivirus vector. (**a**) Displayed are histograms of *CH25H* expression level in HL-60-CV (CV) and HL60-*CH25H*shRNA (shRNA) treated with or without DAC for 4 days, respectively. (**b**) Shown is the survival (%) from DAC-induced HL-60 cell death by *CH25H*-knockdown. HL-60-CV cells (CV) and HL-60-*CH25H*shRNA cells (shRNA) were treated daily with DAC (0–500 nM) for 3 to 4 days, and the change in the cell number was evaluated by MTT assay. (**c**) HL-60-CV cells (CV) and HL-60-*CH25H*shRNA cells (shRNA) were treated with different concentrations (0 and 500 nM) of DAC for 96 h, and apoptosis was assessed by flow cytometry using annexin V and propidium iodide (PI) staining. Positive staining for annexin V or for annexin V and PI show early or late apoptosis, respectively. The value of right area indicates the percentage of the cells in early and late apoptosis. (**d**) Displayed are the histograms of *CH25H* expression level in MDS-L-CV (CV) and MDS-L-*CH25H*shRNA (shRNA) treated with or without DAC for 4 days, respectively. (**e**) Shown is the survival (%) from DAC-induced MDS-L cell death by *CH25H*-knockdown. MDS-L-CV cells (CV) and MDS-L-*CH25H*shRNA cells (shRNA) were treated daily with DAC (0–100 nM) for 4 days, and the change in the cell number was evaluated by MTT assay. The data shown are the average ± SD of three (**a,d**) or five (**b,e**) independent experiments. *p < 0.05; **p < 0.01.

To further examine the role of *CH25H*, we established *CH25H*-knockdown HL-60 clones (HL-60-*CH25H*shRNA) and *CH25H*-knockdown MDS-L clones (MDS-L-*CH25H*shRNA) by transfecting *CH25H*-shRNA lentiviral vector into each cell line, and simultaneously established the control clones transfected with the control vector (HL-60-CV and MDS-L-CV). *CH25H*-knockdown clones revealed a drastic decrease in *CH25H* mRNA expression (the copy ratio of *CH25H* to *RPL27* after DAC treatment was 410 ± 40 in HL-60-CV and 0.5 ± 0.1 in HL-60-*CH25H*shRNA: p < 0.01) (Fig. 4a.).

Both HL-60-CV and HL-60-*CH25H*shRNA cell clones were treated daily with DAC for 3 or 4 days, and the effect of *CH25H*-knockdown on DAC-induced growth suppression was examined by MTT assay. As shown in Fig. 4b, DAC-induced cell death was partially but significantly rescued by *CH25H*-knockdown. The percentage of growth inhibition by DAC treatment (100 nM and 500 nM) was 48.9 ± 1.3% and 24.9 ± 2.3% in HL-60-CV clones; 57.9 ± 2.1% and 35.6 ± 0.8% in HL-60-*CH25H*shRNA clones, respectively on day 3 (p < 0.01); 15.0 ± 0.6% and 4.7 ± 0.6% in HL-60-CV clones; 30.3 ± 1.2% and 13.0 ± 0.7% in HL-60-*CH25H*shRNA clones, respectively on day 4 (p < 0.01).

Apoptosis was detected using flow cytometry by positive staining for annexin V (early apoptosis) or for annexin V and propidium iodide (late apoptosis). The percentage of annexin V(+) and PI (−) and annexin V(+) and PI (+) in DAC (500 nM)-treated HL-60-*CH25H*shRNA clones was decreased as compared with that of HL-60-CV (Fig. 4c). DAC-induced cell death in MDS-L was also partially but significantly rescued by *CH25H*-knockdown (Fig. 4d,e). Taken together, *CH25H*-knockdown by shRNA lentiviral transfection partially protected these cell lines from DAC-induced cell death.

### Exogenous addition of 25-OHC suppresses the growth of MDS/leukemia cell lines *in vitro*

**Figure 5:**
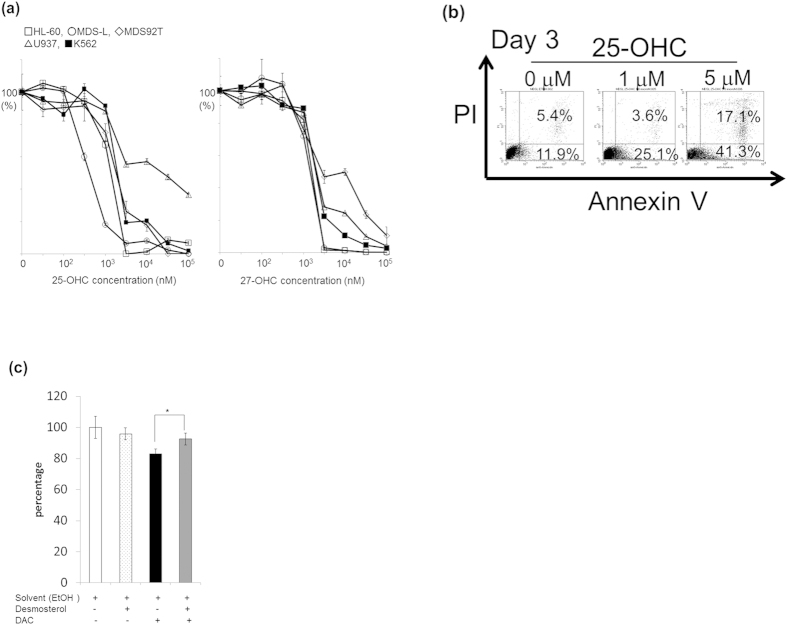
Growth suppressive effect of 25-hydroxycholesterol (25-OHC) on MDS/leukemia cell lines. (**a**) The indicated cell lines were cultured for 3 days in the presence of various concentrations of 25-OHC or 27-OHC, and growth suppression was evaluated by MTT assay. (**b**) MDS-L cells were treated with 0, 1 or 5 μM of 25-OHC for 72 h, and apoptosis was assessed by flow cytometry using annexin V and PI staining. Positive staining for annexin V or for annexin V and PI show early or late apoptosis, respectively. The value of lower right area and upper right area indicate the percentage of cells in early apoptosis and late apoptosis, respectively. (**c**) MDS-L cells were treated with 5 nM DAC in the presence or absence of 10 μM desmosterol for 4 days. Shown is the survival (%) from DAC-induced MDS-L cell death by desmosterol. The change in the cell number was evaluated by MTT assay. The data shown are the average ± SD of five independent experiments and there was significant difference between DAC-treated and DAC plus desmosterol-treated cells. *p < 0.05.

As above described, we could not detect the obvious production of 25-OHC in the cell lysates nor in the culture supernatants of DAC-treated cells. Therefore, to know whether 25-OHC actually suppresses cell growth *in vitro*, we added several concentrations of 25-OHC to the MDS/leukemia cell lines *in vitro*. We also used another oxysterol, 27-OHC as a comparative experiment. MDS-L cells were found to be most sensitive to 25-OHC, with reduced cell viability observed at around 500 nM (Fig. 5a). Half-maximal inhibitory concentration (IC_50_) values of 25-OHC and 27-OHC on the cell lines are shown in Table 2. Except for U937, these cell lines were susceptible to 25-OHC rather than 27-OHC (Table 2). In addition, to examine whether growth suppression by 25-OHC was due to the apoptosis, we performed annexin V and PI staining with flow cytometer. The percentage of annexin V(+) and PI (−) and annexin V(+) and PI (+) in 25-OHC (1 and 5 μM) -treated MDS-L cells was increased as compared with that of untreated MDS-L cells (Fig. 5b).

To examine whether the inhibition of CH25H enzymatic activity modifies its growth suppressive effect, we used a CH25H inhibitor, desmosterol. MDS-L cells were cultivated with 5 nM DAC and/or 10 μM desmosterol for 4 days and cell growth was measured by MTT assay. In the presence of desmosterol, DAC-induced cell growth suppression was rescued to a limited extent but significantly (n = 5, p < 0.05) (Fig. 5c).

## Discussion

In the present study, by gene expression profiling and genome-wide DNA methylation analysis, we identified 13 genes whose mRNA expression was up-regulated more than 20-fold and whose CpG islands at around 1000 bp upstream sequence of the transcription initiation site was hypomethylated more than 10% after DAC treatment as compared with control. Out of these genes we focused on *CH25H* as the gene which seemed to be associated with cell proliferation, apoptosis and cell death (Table 1).

CH25H is an enzyme to convert cholesterol into 25-OHC, one of the oxysterols and the *CH25H*-oxysterol pathway is an entrance to the bile acid synthesis. The sterol-regulatory element binding proteins (SREBPs) are transcription factors involved in the synthesis of cholesterol^40^,^41^. Twenty-five-OHC inhibits SREBPs, and regulates 3-hydroxy-3-methylglutaryl coenzyme A reductase (HMGCR), the rate-limiting enzyme of *de novo* cholesterol synthesis^42^,^43^. In addition, 25-OHC regulates cholesterol homeostasis through a ligand of liver X receptor^44^,^45^. It was reported that 25-OHC has a growth inhibitory effects on various cells^19^,^20^,^21^,^22^. Banker, DE *et al.* reported that the cholesterol level is abnormally increased in AML cells exposed to chemotherapeutic drugs *in vitro* and that blocking these acute cholesterol responses facilitates cell death^46^,^47^. Zhang F, *et al.* reported that DAC inhibits leukemic cell growth through modulating endogenous cholesterol biosynthesis^48^. As shown in Fig. 1 and Supplementary Figure S1, GSEA indicated that lipoprotein and bile acid metabolism pathways are up-regulated by DAC treatment and that these affected gene sets contained *CH25H* and its downstream genes, *CYP7B1* and *HSD3B7* as clearly activated genes (Supplementary Figure S1). These data demonstrate the activation of the *CH25H*-oxysterol pathway by DAC treatment in MDS-L cells.

We hypothesized that up-regulation of *CH25H* after DAC treatment promotes the accumulation of endogenous 25-OHC and it contributes in part to DAC-induced cell death. DAC promoted the expression of *CH25H* in various MDS/leukemia cell lines, particularly in MDS-L (Fig. 2). AZA also induced *CH25H* mRNA expression at high concentrations in HL-60 and MDS-L cells (Fig. 2a–c). To confirm intracellular increase in the enzymatic activity of CH25H, we attempted to detect 25-OHC within cell lysates or culture supernatants of DAC-treated and untreated MDS-L cells with LC/MS and TOF/MS, and in addition with GC-MS. Although the production level of 25-OHC seemed to be quite low, we could detect a small but significant amount of 25-OHC in a larger-scale cell sample after DAC treatment.

Unexpectedly, *CH25H* mRNA expression was induced not only by DNMT inhibitors but also by a similar antimetabolite compound, cytarabine (Fig. 2g). Because it is unlikely that cytarabine acts as a hypomethylating agent, cytarabine might up-regulate *CH25H* mRNA expression by another mechanism. Hence, induction of *CH25H* expression itself might not be a specific phenomenon induced by DNMT inhibitors but rather a general intracellular event induced by cytarabine. Anti-cancer agents exert various cytotoxic actions. From our data we speculate that activation of *CH25H*-mediated pathway is one of their important action mechanisms and that DNMT inhibitors trigger such a universal death signaling as a result of DNA hypomethylation. Further knockdown or overexpression experiments of *CH25H* gene would serve for elucidating the implication of this gene for the therapeutic targets of cancer chemotherapy.

We paid attention to the promoter region of *CH25H* gene and performed the methylation analysis on 1700 bp upstream sequence (divided into F1 to F6 regions) of *CH25H* transcription initiation site. In untreated MDS-L and HL-60 cells, most CpG sites at F1, F2, F5 and F6 were methylated, whereas those of F3 and F4 remained unmethylated (Fig. 3b,c). On the contrary, in DAC-treated cells, a considerable part of CpG sites at F2, F5 and F6 were demethylated (Fig. 3b,c). In particular, release of F6 region from hypermethylated status seems to be important for the recovery of *CH25H* expression. We next evaluated the methylation status of *CH25H* in CD34-positive progenitor cells derived from bone marrow of five healthy donors. Strikingly, CpG sites at F5 and F6 in normal bone marrow CD34-positive cells were hardly methylated. These data strongly suggest that: first, the CpG islands at the promoter region of *CH25H* are hypermethylated and the expression of *CH25H* is suppressed in MDS/leukemia cell lines; second, the promoter region of *CH25H* is hardly methylated and *CH25H* message is expressed to some extent in normal bone marrow CD34-positive cells; and third, DNMT inhibitors promote *CH25H* expression by altering the hypermethylated status of this gene and induce cell death.

Considering our results that the promoter region of *CH25H* was hypermethylated in the blastic subline, MDS-L as compared with that of parental MDS92, we speculate that hypermethylation of the promoter region of *CH25H* is relevant to more aggressive cell growth. To verify this hypothesis on primary MDS/leukemia cells, we investigated the methylation status of *CH25H* F6 region in 4 MDS/AML patients and found one case (UPN9) whose *CH25H* F6 region was hypermethylated (Supplementary Figure S2), although *CH25H* mRNA expression was not examined in this case. About two low-risk MDS cases (UPN1 and UPN2), we verified increased expression of *CH25H* together with hypomethylation of *CH25H* F6 region (Fig. 3f and Supplementary Figure S2). Nonetheless, the present data are indeed insufficient to certify the relation of the methylation status of *CH25H* gene and its expression in primary MDS/leukemia cells. Further information should be accumulated about the data of *CH25H* gene in MDS/leukemia cases and the effects of DNMT inhibitors on the methylation status of *CH25H* as well as clinical outcome.

Although they were cell line-limited studies, we attempted to confirm the close relation of *CH25H* expression to the suppressive effect on leukemic cell growth by several ways. First, *CH25H*-knockdown by transfection of shRNA lentiviral vector in HL-60 and MDS-L cells partially protected the cells from DAC-induced cell death (Fig. 4a–e). Second, exogenous addition of 25-OHC suppressed the growth of MDS/leukemia cell lines *in vitro* (Fig. 5a,b). Third, a CH25H inhibitor, desmosterol rescued the cells from DAC-induced cell growth suppression to a limited extent but significantly (Fig. 5c). These experimental data are of help as evidence that epigenetic silencing of *CH25H* leads to leukemic cell growth and that hypomethylating agents exert their anti-leukemic action partly via activation of the *CH25H*-oxysterol pathway. In addition to this hypomethylating effects, there may exist other underlying action mechanisms of DNMT inhibitors, judging from our observation that DAC also exerted some cytotoxic effect on normal CD34-positive cells although their *CH25H* promoter region was hardly methylated.

The present study raises a possibility that DNMT inhibitors activate the *CH25H*-oxysterol pathway by their hypomethylating mechanism and induce leukemic cell death. Further investigations of the promoter analysis of *CH25H* gene and the therapeutic effects of DNMT inhibitors on MDS and AML will be warranted.

### Support and Financial Disclosure Declaration

Tohyama K received research funding from Eisai Co., Ltd. The other authors have no competing financial interests to declare

## Additional Information

**How to cite this article**: Tsujioka, T. *et al.* Five-aza-2'-deoxycytidine-induced hypomethylation of *cholesterol 25-hydroxylase* gene is responsible for cell death of myelodysplasia/leukemia cells. *Sci. Rep.* **5**, 16709; doi: 10.1038/srep16709 (2015).

## Supplementary Material

Supplementary Information

## Figures and Tables

**Table 1:** Thirteen genes whose expression was up-regulated and whose promoter region was demethylated after DAC treatment.

	Expression ratio	CpG methylation	
	(DAC/control)	−1 kbp	−3 kbp
*CH25H*	188.5	−45%	−38%
*CGA*	154.5	−14%	−14%
*ANGPT2*	63.7	−24°%	−15%
*AQP1*	55.8	−13%	−16%
*CD36*	40.9	−14°%	−17%
*S100A16*	40.3	−18%	−17%
*F2RL2*	37.8	−34%	−16%
*ADD2*	34.1	−22%	−16%
*APOC1*	29.4	−25%	−21%
*MS4A3*	29.1	−15%	−14%
*BHLHE22*	27.4	−19%	−14%
*ALB*	24.9	−12%	−10%
*ABCC3*	20.5	−26%	−23%

**Table 2:** IC50 of 25-OHC or 27-OHC in MDS/leukemia cell lines.

Cell line	25-OHC: IC50±SD (μmol/L)	27-OHC: IC50±SD (μmol/L)
HL-60	1.5 ± 0.08	1.9 ± 0.01
MDS-L	0.6 ± 0.02	1.7 ± 0.01
MDS92T	2.4 ± 0.26	4.1 ± 0.32
U937	4.0 ± 0.60	2.8 ± 0.29
K562	2.5 ± 0.12	2.6 ± 0.02
