# Supplementary material for: Five-aza-2′-deoxycytidine-induced hypomethylation of cholesterol 25-hydroxylase gene is responsible for cell death of myelodysplasia/leukemia cells
Source: Sci Rep. 2015 Nov 18;5:16709. doi: 10.1038/srep16709 (PMC4649363; doi:10.1038/srep16709)
Supplement: Supplementary Information [file srep16709-s1.doc]

**Five-aza-2’-deoxycytidine-induced hypomethylation of *cholesterol 25-hydroxylase* gene is responsible for cell death of myelodysplasia/leukemia cells**

Takayuki Tsujioka^1^, Akira Yokoi^2^, Yoshitaro Itano^3^, Kentaro Takahashi^2^, Mamoru Ouchida^4^, Shuichiro Okamoto^1^,Toshinori Kondo^5^, Shin-ichiro Suemori^1^, Yumi Tohyama^6^, Kaoru Tohyama^1^

^1^Department of Laboratory Medicine, Kawasaki Medical School, Okayama 701-0192, Japan

^2^Eisai Co., Ltd., Tsukuba, Ibaraki, 300-2635, Japan

^3^Department of Anesthesiology, Kawasaki Medical School, Okayama 701-0192, Japan

^4^Departments of Molecular Genetics, Okayama University Graduate School of Medicine,

Dentistry and Pharmaceutical Sciences, Okayama 700-8558, Japan

^5^Division of Hematology, Department of internal Medicine, Kawasaki Medical School, Okayama 701-0192, Japan

^6^Division of Biochemistry, Faculty of Pharmaceutical Sciences, Himeji Dokkyo University, Hyogo 670-8524, Japan

**Supplementary**

**Materials and methods**

**Genome-wide methylation sequencing analysis (MethylC-seq analysis)**

Genomic DNA was purified from cells using the DNeasy Blood & Tissue Kit (Qiagen, Valencia, CA) according to the manufacturer’s instructions. The DNA samples (10 μg/sample) were sheared into 300-bp fragments using the Covaris S-series (Covaris, Woburn, MA), followed by end repair and ligation of Early Access Methylation Adapter Oligo (Illumina, San Diego, CA) by using the Paired-End DNA Sample Prep Kit (Illumina). The adapter-ligated DNA was subjected to sodium bisulfite treatment using the MethylEasy Xceed Rapid DNA Bisulphite Modification Kit (Human Genetic Signatures, North Ryde, NSW, Australia). Each bisulfite-converted sample was amplified in 8 independent tubes as follows: 2.5 U of PfuTurbo Cx Hotstart DNA polymerase (Stratagene, , La Jolla, CA), 5 μl 10X PfuTurbo Cx reaction buffer, 25 μM dNTPs, 200 nM Primer 1, 200 nM Primer 2 (50 μl final). Thermocycling parameters were: initial denaturation at 95°C for 2 min, 98°C for 30 s, 16 cycles of denaturation at 98°C for 10 s, annealing at 65°C for 30 s, and extension at 72°C for 2 min, followed by a final extension at 72°C for 5 min. PCR reaction products were purified using the QIAquick PCR Purification Kit (Qiagen, Hilden, Germany). The purified products (5 μg/sample) were submitted to Takara Bio Dragon Genomics Center (Yokkaichi, Mie, Japan) for the MethylC-seq. The sequencing was performed with the Single-Read Cluster Generation Kit v4 and TruSeq SBS Kit v5 using the Genome Analyzer IIx system (Illumina)^29-32^.

Raw sequence reads were filtered using FASTX toolkit (http://hannonlab.cshl.edu/fastx_toolkit/index.html) to remain reads having Phred Score of 10 or higher for 95% or more bases. Mapping was conducted in an iterative manner described as follows. Reads were mapped to the human genome (hg19) using Bismark (Krueger and Andrews) with no mismatch. Unmapped reads were mapped again allowing up to 3 mismatches. As low quality bases condensed on the right end portion, reads having mismatches in 10% of the most right end were removed from the mapping results. This procedure was repeated 3 times starting from 100 bp followed by 75 and 50 bp by trimming 25 bp from the right end of unused reads. CpG methylation calls were extracted from the mapping results. Upstream CpG methylation ratio for each gene was estimated by averaging out CpG methylation ratios in the 1 kb (or 3 kb) upstream region from the transcription start site annotated in UCSC refGene.

**Gene expression profiling and the gene set enrichment analysis (GSEA)**

Gene expression profiling of MDS-L cells was examined in triplicate experiments (DAC-treated (4 nM) or untreated cells were harvested on day 7 in triplicate assays independently). Significant functions were calculated by Ingenuity Pathways Analysis (Ingenuity Systems, CA, USA) and DAVID software (National Institute of Allergy and Infectious Diseases (NIAID), NIH) from a list of genes showed 2-fold increase or decrease after DAC treatment. We previously submitted our data to NCBI GEO17, and the study number is GSE39628 (<http://www.ncbi.nlm.nih.gov/geo/query/acc.cgi?acc=GSE39628>)^17^.

The gene set enrichment analysis (GSEA) was performed by the use of the gene expression profiling data as above obtained and by handling the GSEA software and the Molecular Signatures Database according to the references ^35,36^ .

# Ultraperformance liquid chromatography coupled with Q-TOF mass spectrometry (UPLC/Q-TOF MS) and gas chromatography-mass spectrometry (GC-MS)

As for UPLC/Q-TOF MS, DAC-treated or untreated cells were lysed by a standard LC-MS method and liquid chromatography was performed on Waters Acquity UPLC™ system (Waters, Manchester, UK), using the Acquity UPLC HSS T3 column (2.1×100 mm, 1.8 μm; Waters, Manchester, UK). The column oven temperature was maintained at 40°C. The mobile phases were (A) 0.1% formic acid in water and (B) acetonitrile. The linear gradient program began with 30 % (A) for 20 min and proceeded to 100 % (B) over 20 min and furthermore maintained to same conditions (100 %: B), The total cycle time was 45 min, with a flow rate of 0.2 ml/min and an injection volume of 5 μL.

Mass spectrometry was performed using Waters Xevo QTOF MS (Waters Corp., Manchester, UK). Ionization was performed in the positive electrospray (ESI+) mode. The mass range was set at m/z 50–1,000 daltons. The conditions used for the ESI source were as follows: capillary voltage, 3.0 kV; sampling cone, 15 V ^38, 39^ .

As for GC-MS, sterol fraction was extracted from DAC-treated or untreated cells with n-hexane. Such a fraction retained onto the Bond Elut SI cartridge (Agilent Technologies Inc., California) were eluted, dried and derivatized with trimethylsilyl (TMS) reagent. The sample was re-suspended in n-hexane for GC–MS analysis. GC–MS analysis was conducted (GCMS-TQ8030; Shimadzu Corp., Kyoto) using an Rxi-5ms capillary column (30 m×0.25 mm, 0.25 μm thickness, DB-5 ms; Restek Corp., Pennsylvania). The oven temperature program was set as follows:180 °C for 1 min, 20 °C/min to 250 °C and then 5 °C/min to 300 °C where the temperature was kept for 11 min. Helium was used as a carrier gas and the flow rate was set at 37.7 cm/sec. Injection was performed in the splitless mode and the MS ion source temperature and interface temperature were 200 °C and 250 °C, respectively. Samples (1 μl) were injected for analysis. The mass spectrometer was operated in the selected ion monitoring mode. A few ions were detected simultaneously. The ions used for analysis (m/z) for the compounds were as follows: 24-hydroxycholesterol, 159; 25-hydroxycholesterol, 129; 27-hydroxycholesterol, 456.

**Supplementary Table S1.** **The primer sequences in the promoter region of *CH25H***

**Supplementary Figure S1. Heat map presentations of *CH25H*-related pathways in the gene set enrichment analysis (GSEA)**

DAC-treated (Decitabine) or untreated (Control) MDS-L cells were harvested on day 7 in triplicate assays and the gene expression profiling data were used for GSEA by handling the GSEA software and the Molecular Signatures Database according to the references ^(35,36)^. Two gene sets strongly up-regulated by DAC treatment are presented as heat map presentations: (a) metabolism of lipids and lipoproteins, and (b) bile acid and bile salt metabolism. In (a), the top 50 activated genes out of 395 genes included in the gene set are shown. In (b), total 22 genes in the gene set are shown. Both gene sets contain *CH25H* as the most activated gene by DAC treatment and its downstream genes, *CYP7B1* and *HSD3B7*.

**Supplementary Figure S2. The methylation analysis in the F6 promoter region of *CH25H* in primary MDS/ leukemia CD34-positive progenitor cells**

We analyzed methylation status of independent plasmid clones derived from CD34-positive cells of primary MDS and leukemia patients (● methylated cytosine, 〇 unmethylated cytosine). The primary cases were categorized by WHO classification as follows: UPN1 (MDS: Refractory anemia,), UPN2 (MDS: Refractory cytopenia with multilineage dysplasia), UPN9 (MDS: Refractory anemia with excess blasts-1), UPN10 (AML: M1).
